# Differential Regulation of NK Cell Receptors in Acute Lymphoblastic Leukemia

**DOI:** 10.1155/2022/7972039

**Published:** 2022-05-23

**Authors:** Le Jie Lee, Norfarazieda Hassan, Siti Zuleha Idris, Suresh Kumar Subbiah, Heng Fong Seow, Norhafizah Mohtaruddin, Kian Meng Chang, Raudhawati Osman, Hishamshah Mohd Ibrahim, Sheila Nathan, Maha Abdullah

**Affiliations:** ^1^Department of Pathology, Faculty of Medicine & Health Sciences, Universiti Putra Malaysia, 43400 UPM Serdang, Selangor, Malaysia; ^2^UPM-MAKNA Cancer Research Laboratory, Institute of Bioscience, Universiti Putra Malaysia, Serdang 43400 UPM, Selangor, Malaysia; ^3^Regenerative Medicine Cluster, Advanced Medical and Dental Institute, Universiti Sains Malaysia, 13200 Kepala Batas, Penang, Malaysia; ^4^Department of Hematology, Hospital Ampang, Clinical Hematology Laboratory, Jalan Mewah Utara, Pandan Mewah, Ampang, Selangor 68000, Malaysia; ^5^Hematology Unit, Hospital Kuala Lumpur, Jalan Pahang, 50586 Wilayah, Persekutuan Kuala Lumpur, Malaysia; ^6^Pediatric Department, Hospital Kuala Lumpur, 50586 Wilayah, Persekutuan Kuala Lumpur, Malaysia; ^7^School of Biosciences and Biotechnology, Faculty of Science and Technology, Universiti Kebangsaan Malaysia, 43600 UKM Bangi, Selangor, Malaysia

## Abstract

Cancer immunotherapies are preferred over conventional treatments which are highly cytotoxic to normal cells. Focus has been on T cells but natural killer (NK) cells have equal potential. Concepts in cancer control and influence of sex require further investigation to improve successful mobilization of immune cells in cancer patients. Acute lymphoblastic leukemia (ALL) is a hematological malignancy mainly of B cell (B-ALL) and T cell (T-ALL) subtypes. Influence of ALL on NK cell is still unclear. Targeted next-generation sequencing was conducted on 62 activating/inhibitory receptors, ligands, effector, and exhaustion molecules on T-ALL (6 males) and normal controls (NC) (4 males and 4 females). Quantitative PCR (q-PCR) further investigated copy number variation (CNV), methylation index (MI), and mRNA expression of significant genes in T-ALL (14 males), NC (12 males and 12 females), and B-ALL samples (*N* = 12 males and 12 females). Bioinformatics revealed unique variants particularly rs2253849 (T>C) in *KLRC1* and rs1141715 (A>G) in *KLRC2* only among T-ALL (allele frequency 0.8-1.0). Gene amplification was highest in female B-ALL compared to male B-ALL (*KLRC2*, *KLRC4*, and *NCR3*, *p* < 0.05) and lowest in male T-ALL cumulating in deletion of *KLRD1* and *CD69*. MI was higher in male ALL of both subtypes compared to normal (*KIR2DL1-2* and *4* and *KIR2DS2* and *4*, *p* < 0.05) as well as to female B-ALL (*KIR3DL2* and *KIR2DS2*, *p* < 0.05). mRNA expressions were low. Thus, ALL subtypes potentially regulated NK cell suppression by different mechanisms which should be considered in future immunotherapies for ALL.

## 1. Introduction

Conventional cancer treatments such as chemotherapy and radiotherapy are highly cytotoxic, induce severe side effects, and in many instances are not effective. Thus, in the last decades, efforts were made in development of more targeted therapies including cell therapies, particularly to reestablish the function of the immune system. Cancer cells evade immune attack at various levels, by downregulating human leukocyte antigen (HLA) and antigen presentation or promote cold tumor environment by inducing regulatory and suppressor cells such as regulatory T cells, M2 macrophages, or immunosuppressive dendritic cells. Cancer cells are also able to inhibit immune cells at a state of senescence or exhaustion through death receptors such as Fas or PD-1/CTLA-4/LAG-3, respectively. The aim of immunotherapy is to remove these blockades, whether using molecular or cellular means to release immune cells back into full capacity to specifically kill tumor cells. These have focused on manipulation of T cells. Immune checkpoint inhibitors, monoclonal antibodies, cancer vaccines, cell adoptive therapies, and more recently chimeric antigen receptor- (CAR-) T cells [1] have reached clinical trials but results are not completely satisfactory.

NK cells of the innate system kill cancer cells directly, without the need for antigen sensitization and rapidly activate the adaptive system through cytokine secretion. NK cells respond to targets cells on the missing self-concept from loss of cytolytic inhibition in the absence of self-ligand (MHC-class I molecules) interaction with NK cell inhibitory receptors. This contrasts with cytotoxic CD8+ T cells which bind to specific antigen on MHC-class I for activation. NK receptors genes are clustered at two distinct sites, the natural killer gene complex (NKC) and the leukocyte receptor complex (LRC). NKC encodes the C-type lectin-like superfamily such as NKG2D and CD94/NKG2x while LRC-codes for Ig-like natural cytotoxicity receptor (NCR) and killer cell Ig-like receptors (KIR). The three types of NCR are NKp46 (NCR1), NKp44 (NCR2), and NKp30 (NCR3), which contribute to NK cell tolerance to self-tissues [2]. NK cells are inhibited by signaling of receptors bearing the immunoreceptor tyrosine-based *inhibitory* motif (ITIM), while activation is mediated by immunoreceptor tyrosine-based *activation* motif (ITAM). Signaling lymphocytic activation molecule (SLAM) family such as self-ligand SLAM6-7 and non-self-ligand CD244 is expressed on hematopoietic cells in chronic infection and cancer. The expression of these molecules signifies the exhausted phenotype characterized by loss of proliferative capacity and inability to produce cytokines and exerts killing [3] which are potential targets for cancer immune escape. NK cells undergo a licensing process to become educated and fully competent, where NKG2A or inhibitory KIRs (iKIRs) interact with cognate MHC class-I (HLA-I) ligands. NK cells lacking iKIR are unlicensed and impaired in response to stimulation. Nevertheless, unlicensed NK cells may still function under specific conditions [4]. Effector cytolytic activity is mediated by perforin and granzyme and death receptors, Fas and TRAIL, to induce cell apoptosis. Stress from viral infection or tumor development induces DNA damage response, senescence program, or tumor suppressor genes to upregulate ligands that bind activating receptors in NK shifting the balance toward NK cell activation and elimination of target cell. Finally, inhibitory and activating signals integrate to render NK cell either functional or nonfunctional.

The missing self-concept in potential CAR-NK cells is advantageous over CAR-T cells as it has in addition intrinsic killing mechanism, minimizes graft-versus-host-disease, and has more potential to induce alloreactivity towards recipient cancer cells from allogeneic donors with mismatched ligands. CAR-NK cell has become an alternative therapy option as it also incurs fewer side effects post transplantation [1]. NK cells express traditional immune checkpoint molecules such as PD-1, TIM-3, and TIGIT. Therefore, NK cells provide an alternative cell source with similar effector function but decreased toxicity and the potential for an “off-the-shelf” model [5]. Many studies have shown beneficial effect of T and NK cell immunotherapy to cancer patients; however, outcomes for some patients are still suboptimal, and strategies to overcome barriers to success are much needed [5].

Acute lymphoblastic leukemia (ALL) is a malignancy of immature lymphoblasts originating from the bone marrow and spreading to the blood. It is the most common cancer (25%) in children (ages 0-14) but accounts for <1% of adult cancers. Approximately 20% leukemia is adults [6]. The majority of ALL are of the B-ALL subtype and only 15% T-ALL. There is a predominance of males over females. Male/female (M/F) ratio among B-ALL ranges from 1.18 in Morocco [7] to 1.69 in South China [8]. M/F ratio for T-ALL is significantly higher, 2.19 among children in Nordic countries [9], 2.93 in Morocco [7], and 3.05 in South China [8].

The presence of NK cells in bone marrow of ALL significantly increased patient survival [10]. NK cell-mediated cytotoxicity was significantly reduced in ALL patients compared to controls and reduced in high-risk ALL compared to standard-risk patients [11]. Advances in adaptive NK cell therapy based on selective expansion of NK cells with stronger cytotoxicity are expected to further improve survival in resistant ALL [12]. Thus, NK cells are important in immunity against ALL and the relevant mechanism of immune escape should be identified. We aim to determine genetic and epigenetic variation in NK cells among ALL subgroups in order that immunotherapies are established for the correct target.

## 2. Materials and Methods

### 2.1. Sample Selection

Bone marrow and peripheral blood samples were collected from Hematology Unit, Hospital Kuala Lumpur, between May 2011 and March 2013. Patient demography is shown in Supplementary Table S1. Diagnoses of leukemia cases were determined by hematopathologists according to FAB (1976) and WHO Classification 2016 guidelines based on morphology, cytochemistry staining, and immunophenotyping results. Approval to conduct the study was obtained from the Medical Research and Ethics Committee, Ministry of Health, Malaysia. All procedures were in compliance with the Helsinki Declaration on ethical principles for medical research. Informed consents were obtained from participants.

### 2.2. Study Group

Only confirmed B-ALL and T-ALL cases were selected excluding myeloid, mix-phenotypic, or chronic leukemia cases. Male and female of adult and pediatric cases were included. Lastly, samples with percentage of lymphoblast less than 20%, lysed or without a diagnosis, were excluded.

Screening for NK-related genes using next-generation sequencing (NGS) was conducted on 8 healthy individuals (4 males and 4 females) and 6 T-ALL patients. Further testing on copy number variation was performed on 24 healthy individuals (12 males and 12 females), 24 B-ALL patients (12 males and 12 females), and 10 T-ALL male patients. During this period, only two T-ALL female patients were diagnosed and were excluded.

### 2.3. DNA Extraction

DNA from approximately 1.5 × 10^6^ mononuclear cells (Ficoll-Paque PLUS, GE Healthcare, Sweden) isolated from blood or bone marrow samples was extracted by the salting out method [13] following overnight incubation with proteinase K (20 mg/mL) (Finnzymes, Finland) and dissolved in TE buffer.

### 2.4. Next-Generation Sequencing

SureSelect^XT^ target enrichment system for Illumina paired-end sequencing custom kit (Agilent Technologies, USA) was used to perform target enrichment for genes of interest. Agilent SureDesign (https://earray.chem.agilent.com/suredesign) was used to design the probes. A total of 62 natural killer (NK) cell associated receptors (*KIR*, *KLR*, and *NCR*), *KLR* ligands, cytotoxic effector (*TNF*, *FAS*, and *GZM*), and exhaustion molecules (*SLAM* family) were selected (Supplementary Table S2). UCSC Genome Browser (https://genome.ucsc.edu) provided details of genes on chromosome, start position, and end position based on human genome 19 (Hg 19) assemblies released in February 2009. Databases of RefSeq, Ensembl, CCDS, Gencode, and VEGA were included. The entire transcribed region with 10 bases from 3' end and 10 bases from 5' end was included. For probe tiling parameters, 2x tiling density with least stringent masking and balanced boosting was selected. Probes were ordered and synthesized by Agilent Technologies, USA.

DNA was quantified using the Qubit® dsDNA assay kit (Life Technologies, USA) according to manufacturer's instructions. Three *μ*g of nondegraded genomic DNA (gDNA) was sheared in microTUBE AFA Fiver Snap-Cap with Covaris M220 Focused-ultrasonicator (Covaris, USA), into small fragments of 150 bp to 200 bp. Optimal conditions were at 75 W of peak incident power, 20% duty factor, 200 cycles per burst, 350 s of treatment time, and temperature at 20°C. Sheared gDNA were purified using AMPure XP beads (Beckman Coulter Genomics, USA) followed by quality assessment with 2100 Bioanalyzer (Agilent, USA).

SureSelect Library Prep Kit, ILM (Agilent Technologies, USA), was used to repair the overhang 3' and 5' ends of sheared DNA and incubated at 20°C for 30 min on a thermal cycler and purified using AMPure XP beads and eluted with nuclease-free water. “A” bases were added to the 3' end of DNA fragment to create “A” base overhang with dATP and Exo(-) Klenow (Agilent Technologies, USA) in a thermal cycler and again purified. Ligation of the indexing-specific paired-end adaptor was performed at molar ratio 10 : 1 of adaptor to genomic DNA inserts using SureSelect adaptor oligo mix and subsequently purified. The adaptor-ligated library was then amplified with the SureSelect Library Prep Kit, ILM, and SureSelect Target Enrichment Kit ILM Indexing. Polymerase chain reaction (PCR) was carried out using DNA Engine Tetrad® 2 thermal cycler (Bio-Rad, USA) with PCR programme: 98°C for 2 minutes, 98°C for 30 seconds, 65°C for 30 seconds, and 72°C for 1 minute for 5 cycles followed by final extension at 72°C for 10 min. Following purification, amplified library was assessed on a Bioanalyzer with DNA1000 chip. Electropherogram with peak size distribution from 250 bp to 275 bp was expected.

For hybridization, SureSelect Hyb buffer, SureSelect RNase block, SureSelect block mix, and SureSelect capture library were prepared per manufacturer's instructions. Concentrated prepped library with SureSelect block mix was first denatured at 95°C for 5 min. Final mixture was incubated at 65°C for 16 hours.

Hybrid capture was performed with Dynabeads MyOne Streptavidin T1. Hybridization mixture was added and incubated on a Nutator for 30 min at RT. Further wash steps were according to manufacturer's protocol. Finally, captured DNA retained on the Streptavidin beads was mixed with nuclease-free water.

The captured library was amplified to add index tags. The nucleotide sequence in each of the index is listed in Supplementary Table S3. Thermal cycle was according to programme in Supplementary Table S4. Reaction mix was purified using Agencourt AMPure XP beads and eluted with nuclease-free water. The quality of amplified captured library with index tags was assessed by High Sensitivity DNA assay using 2100 Bioanalyzer (Agilent Technologies, USA). Electropherogram with peak size distribution approximately from 300 bp to 400 bp was expected. The concentration of sample was determined by integration under the peak.

KAPA library quantification kit Illumina platform (KAPABiosystem, USA) was used to assess the quantity of index-tagged library. PCR amplification was performed using Mastercycler® ep realplex (Eppendorf, USA). PCR cycle profiles consisted of initial denaturation at 95°C for 5 min followed by 30 cycles at 95°C at 30 s and 60°C at 45 s. Absolute quantification against the standard curve was used to calculate the concentration of the index-tagged libraries. The average size-adjusted concentration (in pM) for each dilution of every library that was assayed was calculated by multiplying the calculated average concentration with the following factor: size of DNA standard is in bp/average fragment length of library in bp.

TruSeq PE Cluster Kit V3-cBot-HS and instrument cBot (Illumina, USA) were used for cluster generation according to manufacturer's instructions. For sequencing after cluster generation, TruSeq SBS Kit V3-HS (200 cycles) (Illumina, USA) was the sequencing kit while the sequencer was Illumina HiSeq2000.

### 2.5. Bioinformatics Analysis

Avadis NGS v1.6 software (Life Technologies, USA) was used to analyse the output data after next-generation sequencing. The workflow started with quality control and filters followed by raw reads alignment to the reference genome, human genome 19, Hg 19 (UCSC Genome Browser https://genome.ucsc.edu). Small variants and copy number analyses were performed using the same software. In analysis of NGS data to detect small variants, detection algorithms compared the aligned reads against the reference (Hg 19) at each position. A judgment was then made based on the distribution of nucleotides (A, T, C, and G) at that position and the likelihood of error.

### 2.6. Copy Number Variations by Real-Time PCR

Genes with copy number variations detected by NGS were examined by real-time PCR with a larger number of samples as above. Primers for 9 target genes *NCR1*, *NCR3*, *CD69*, *LAIR2*, *KLRC1-C4* and *KLRD1* were designed using Primer3Plus (http://www.bioinformatics.nl/cgi-bin/primer3plus/primer3plus.cgi.). Primer-BLAST (https://www.ncbi.nlm.nih.gov/tools/primer-blast) was used to generate primers. Primer sequences for reference gene and all target genes are listed in Supplementary Table S5. Real-time PCR was conducted with Mastercycler® ep realplex (Eppendorf, Germany). The data was normalized using *GAPDH* as reference gene. Standard curves for each gene were developed using serial dilutions from DNA sample of K562 cell line. Maxima SYBR Green qPCR Master Mix (Thermo Scientific, USA) was used to perform the PCR reaction. The PCR profile is as follows: initial denaturation at 95°C for 5 minutes and then 35 cycles of 1 minute at 94°C, 30 s at 58°C, and 30 s at 72°C, followed by melting curve from 55°C to 95°C with ramping time of 15 minutes. Each sample was run in triplicate.

### 2.7. Bisulfite Conversion

EZ DNA methylation-gold™ kit (Zymo Research, USA) was used for bisulfite conversion of genomic DNA from unmethylated cytosine to uracil according to manufacturer's protocol. The quality and quantity of converted DNA was determined by NanoDrop 1000 spectrophotometry (Thermo Scientific, USA) with setting adjusted to single-stranded DNA.

### 2.8. Quantitative Methylation-Specific PCR

Reference gene *beta-ACTIN* and genes of interest were designed for both pairs of primers for methylated and unmethylated templates. Both sets of primers for reference gene were designed to only amplify bisulfite-converted template specifically. The DNA sequences for promoter regions of target genes were retrieved from database ENSEMBL (http://www.ensembl.org/index/html). The primers were designed using MethPrimer software (http://www.urogene.org/methprimer/) and are shown in Supplementary Table S6. *In silico* validation of designed primers was performed using Oligo Calculator (http://www.basic.northwestern.edu/biotools/oligocalc.html), MFOLD (http://frontend.bioinfo.rpi.edu/applications/mfold/cgi-bin/dna-form1.cgi), and IDT OligoAnalyzer (https://www.idtdna.com/calc/analyzer). The specificities of the designed primers were confirmed by BiSearch software (https://bisearch.enzim.hu).

A serial dilution of bisulfite converted DNA from cell line K562 was done on each gene of interest including the reference gene to generate standard curves, used for quantification. PCR amplification was performed using Mastercycler® ep realplex (Eppendorf, Germany) with Maxima SYBR Green qPCR Master Mix (Thermo Scientific, USA). PCR conditions were initial denaturation step of 15 minutes at 95°C, followed by 40 cycles of 95°C for 15 seconds, 55°C for 30 seconds, and 72°C for 30 seconds. For melting curve, temperature increment from 55°C to 95°C with ramping time of 15 minutes was performed.

### 2.9. Analysis of Real-Time PCR Data

Best *R*^2^ values were used to determine the threshold cycle or C_T_ values using realplex software version 2.2. Standard curves were only accepted when the *R*^2^ values were higher than 0.99. At the same time, melting curve analysis was performed to confirm the specificity of amplification and the appearance of primer dimers. Only one distinct peak should be seen in the melting curve. Relative expression of each gene was determined by using standard curve method. Levels of amplified methylated and unmethylated fragments were normalized to reference *beta-ACTIN*. The methylation index is calculated based on the following formula: [(methylated/reference)/(methylated/reference + unmethylated/reference)] × 100.

### 2.10. Gene Expression

#### 2.10.1. RNA Extraction

Total RNA was extracted from 5 × 10^6^ mononuclear cells using the Tri-Reagent (Molecular Research, USA) according to manufacturer's instructions. Contaminating DNA was degraded with DNase I (New England Biolabs, UK) in the presence of recombinant RNasin ribonuclease inhibitor (Promega, USA) following manufacturer's protocol. Reverse transcription was then performed on 4000 ng of RNA using oligo(dT) 15 primer (Promega, USA) and M-MLV RT (Promega, USA) in the presence of recombinant RNasin ribonuclease inhibitor (Promega, USA).

Real-time PCR amplification was performed with Taq polymerase (Fermentas, USA), on a Mastercycler® ep realplex (Eppendorf, Germany). Reference gene *CD56* was used to normalize the gene expressions. Primer sequences for the genes are listed in Supplementary Table S7.

For *KIR2DL1*, *2DL3*, *2DL4*, *3DL3*, and *2DS4*, experimental validated All-in-One qPCR primers were purchased from GeneCopoeia, USA. The sequences for these primers were not provided.

A serial dilution of cDNA from pooled normal and ALL samples was first conducted on each gene of interest including the reference genes to generate standard curves. PCR was performed with Maxima SYBR Green qPCR Master Mix (Thermo Scientific, USA). The PCR profile is as follows: initial denaturation at 95°C for 5 minutes and then 40 cycles of 1 minute at 94°C, 30 s at 58°C, and 30 s at 72°C, followed by melting curve from 55°C to 95°C with ramping time of 15 minutes. For *KIR2DL1*, *2DL3*, *2DL4*, *3DL3*, and *2DS4*, the annealing temperature was 60°C instead of 58°C. Each sample was run in triplicate.

### 2.11. Statistical Analysis

Statistical analysis was carried out by SPSS version 22. Due to small sample size, nonparametric tests were used. Fisher's exact test was performed to analyse NGS SNPs data. Mann-Whitney *U* test was used to compare CNV between two groups. NGS data for CNV and real-time PCR data were expressed as mean ± standard deviation (SD). *p* value below 0.05 was considered significant.

## 3. Results

### 3.1. Unique Small Variants Were Identified in T-ALL

Small variants are a combination of single (SNP) and multiple nucleotide polymorphism (MNP). SNPs are single nucleotide variation within DNA sequences in a specific locus. MNP refers to the phenomenon where a set of adjacent SNPs occur together. Only small variants with protein effects (stop gained, nonsynonymous coding, splice site, and exonic) were further examined. Small variants in both inhibitory and activating genes were observed in KIR genes (*2DL1*, *3*, and *4*, *2DS4*, and *3DL2-3*), *KLRC1-3*, *KLRD1*, *NCR1-2*, and *LILRB1* (Table 1). *LILRB1* gene had the highest small variants followed by KIR. Normal female and T-ALL male shared more variants among these genes by comparison of either with normal male particularly in *NCR1* and *2*. Unique among females was the high polymorphism in activating *KIR2DS4* with six variants. Of interest was the presence of a single variant in *KLRC1* rs2253849 (T>C) (allele frequency = 1) and another in *KLRC2* rs1141715 (A>G) (allele frequency = 0.8), unique to T-ALL male group. All groups had unique variants in *KLRC3* of which the ones in normal male and female were novel. Details of the unique SNPs and MNPs present exclusively in T-ALL are shown in Table 2 and for normal male and normal female are shown in Supplementary Table S8 and S9, respectively.

### 3.2. Copy Number Amplification Was Associated with B-ALL Females While Gene Deletion Was Observed in T-ALL Males

**Figure 1:**
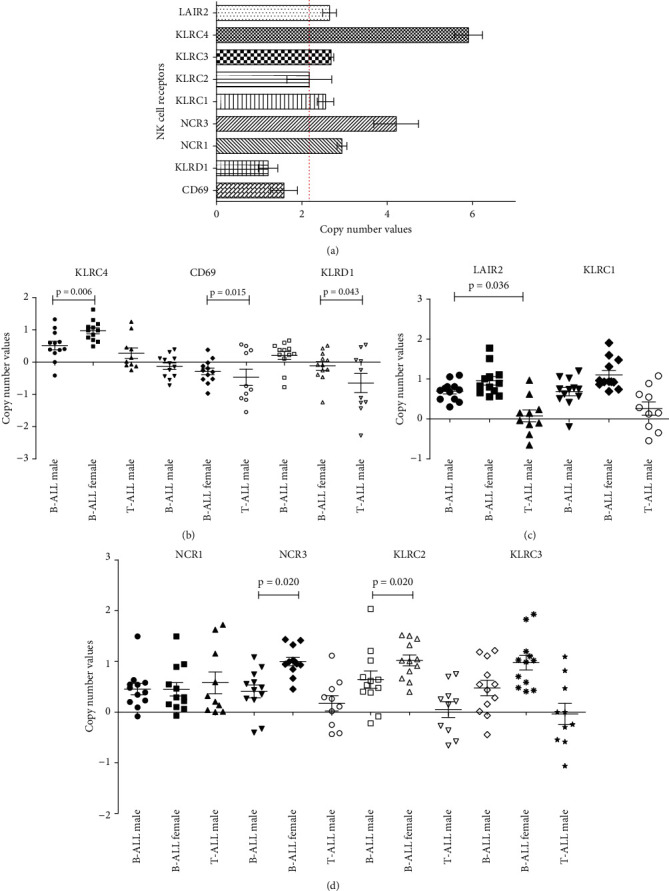
Copy number variations in ALL patient. (a) Copy number amplification/deletion in inhibiting and activating NK receptor in T-ALL male (*n* = 6) normalized to normal male controls (*n* = 4) using NGS method. Broken line indicates normal gene copy number of 2. (b–d) Copy number amplification/deletion of the same genes in T-ALL male (*N* = 10) and B-ALL patients (*N* = 12 males and 12 females) normalized to healthy individuals of same sex (*N* = 12 males and 12 females) using quantitative-PCR method (q-PCR). An initial normalization with reference gene *GAPDH* was performed. Horizontal bars showed *p* values of the Mann-Whitney *U* tests to compare frequencies in copy number amplification between two groups. *p* < 0.05 was considered significant. Data presented in mean ± standard deviation.

Next-generation sequencing detected gene copy number amplification (*LAIR2*, *KLRC1-4*, *NCR1*, and *NCR3*) and deletion (*CD69* and invariant subunit *KLRD1* and CD94) in T-ALL male after normalization to normal male (Figure 1(a)). Gene copy number less than 2 was considered as deletion, while values above 2 were considered as amplification [14]. *KLRD1* forms a complex with activating (NKG2C and NKG2E) as well as inhibitory (NKG2A) receptors. None of the KIR genes showed significant copy number variation (CNV).

Quantitative PCR on CNV performed on 10 T-ALL male patients supported trend in amplification and deletion of the genes in T-ALL males (Figures 1(b)–1(d)). Initial normalization with *GAPDH* was performed for each gene followed by normalization to copy number of normal controls of same sex (*N* = 12 males and 12 females). Normalized copy number values of 1 ± 0.35 were indicative of duplication or amplification, while values −1 ± 0.35 were indicative of deletion. No copy number differences were observed between normal male and female controls as the calculated copy numbers fell in normal range (neither −1 ± 0.35 nor 1 ± 0.35). *CD69* and *KLRD1* were in “deletion” region, while the others were in “amplified” region (Figures 1(b)–1(d)).

In general, CN amplifications were higher in B-ALL female compared to male with significant increase in three genes (*NCR3*, *KLRC2*, and *KLRD4*). Between the ALL subtypes, CNV were higher in B-ALL male compared to T-ALL males, significantly in *LAIR2*, *CD69*, and *KLRD1* with the latter two being deleted. (Comparison between T-ALL male and B-ALL female was not performed.)

### 3.3. Methylation Index Was Significantly Higher in ALL Male

**Figure 2:**
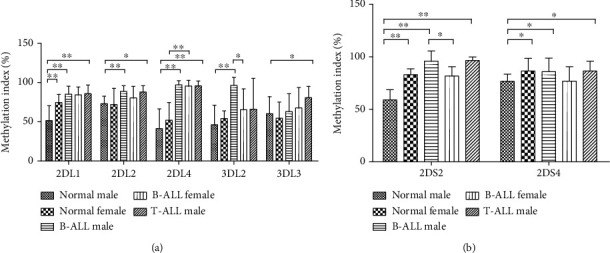
Methylation indexes in the promoter regions of (a) inhibitory KIR genes and (b) activating KIR genes in normal male controls (*n* = 12), normal female controls (*n* = 12), B-ALL male patients (*n* = 12), B-ALL female patients (*n* = 12), and T-ALL male patients (*n* = 10). Data are presented as mean ± standard deviation. Mann-Whitney *U* test compared mean of two groups. “∗” indicates statistical significance *p* < 0.05 and “∗∗” indicates statistical significance *p* < 0.001.

Calculated methylation index (MI) among inhibitory KIR genes (Figure 2(a)) was significantly higher in the promoter region of *KIR2DL1*, *2*, and *4* and *3DL3* in T-ALL male compared to normal male controls. Similarly, methylation indexes of *KIR2DL1*, *2*, and *4* and *3DL2* were significantly higher in B-ALL male patients compared to normal male controls. In contrast, MI in only one gene, *KIR2DL4*, was significantly higher in B-ALL female compared to normal female control. Among normal controls, *KIR2DL1* was significantly higher in female compared to male. Among B-ALL, MI for *KIR3DL2* was significantly higher in male compared to female (Figure 2(b)).

For activating genes (Figure 2(b)), again methylation indexes for both T-ALL male and B-ALL males were significantly higher in *KIR2DS2* and *2DS4* compared to normal male. In normal samples, MI for both activating genes was significantly higher in females. MI of *KIR2DS2* was significantly higher in B-ALL male compared to female.

### 3.4. Gene Expression of NK Cell Receptors Were Not Significant between Sexes

**Figure 3:**
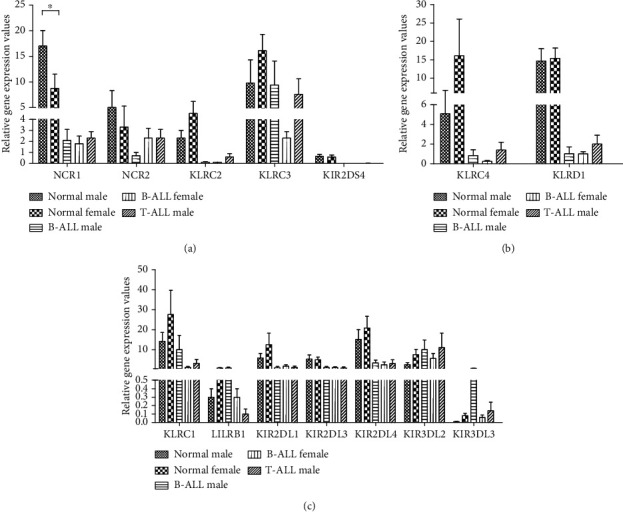
(a–c) mRNA expression of activating receptors in normal male controls (*n* = 12), normal female controls (*n* = 12), B-ALL male patients (*n* = 12), B-ALL female patients (*n* = 12), and T-ALL male patients (*n* = 14) using qPCR. Data are presented as mean ± standard error of mean. Mann-Whitney *U* test compared mean between two groups. “∗” indicates statistical significance *p* < 0.05 and “∗∗” indicates statistical significance *p* < 0.001.

Quantitative PCR determined relative mRNA expressions of selected NK cell receptors in T-ALL male (*N* = 10), B-ALL (*N* = 12 males and 12 females), and normal control (*N* = 12 male and 12 females) after normalization to reference gene *CD56* (Figure 3). Normalization with reference gene *GAPDH* showed no difference in trend or significance (data not shown). Since ALL samples and normal controls were collected from different sites, comparison of gene expression was only performed between sexes of same group. No significant difference was observed for all genes analysed in normal and ALL except significantly higher mRNA for *NCR1* in males compared to females among normal control. The relatively low levels of mRNA in bone marrow samples suggested not suitable sampling site even though potential site for tumor infiltrating of immune cell.

### 3.5. Weak Correlation of Gene Copy Number with Gene Expression Levels

Weak to intermediate negative yet significant correlations were observed in *NCR1* (*r*_*s*_ = −0.359, *p* = 0.023) and *KLRC4* (*r*_*s*_ = −0.319, *p* = 0.045) in combined samples from all groups. Among T-ALL patients however, strong positive correlations were observed for *KLRC2* (*r*_*s*_ = 0.767, *p* = 0.016) and *KLRD1* (*r*_*s*_ = 0.700, *p* = 0.036).

### 3.6. *KIR* Methylation Status Was Negatively Correlated with Gene Expressions Levels

In general, there was none or weak to intermediate negative correlations in all groups of samples. Significant was only observed for *KIR2DL4* (*r*_*s*_ = −0.610, *p* < 0.001).

## 4. Discussion

The idea of immunotherapy is to remove the blockade imposed on immune cells by cancer cells; however, as complex as the immune system is, it is just as complex to overcome this barricade. As knowledge of immune checkpoint blockades advance, it is clear that there are immunosuppressive actions at various levels in immune cell development which function to balance an overactive harmful response to the host. Genetic polymorphism, copy number variation (CNV), and epigenetic changes in methylation index (MI) may impact NK cell-associated genes rendering the individual susceptible to tumor escape and progression. Heterogeneity within cancers such as acute lymphoblastic leukemia (ALL) may have different effect on natural killer (NK) cell immunity.

KIR receptors are highly complex not only as they consist of several inhibitory and activating genes but are not all inherited in an individual. Only framework genes (*KIR2DL4*, *3DL2*, *3DL3*, and *3DP1*) are present in all genomes, while the other genes are present as haplotype A or B. Not all KIR genes inherited are constitutively expressed at mRNA or protein levels. Very few studies have reported NK associated genes in ALL. The results are unclear as KIR genotyping of activating genes (KIR2DS1-5 and 3DS1) in one study revealed significantly lower frequency of only 2DS3 [15] in a Chinese population but all KIR [16] in a white population suggesting ethnicity influence. Another study genotyping *KIR2DS5* gene showed no difference in frequency between controls and acute lymphoblastic leukemia (all *p* > 0.05) [17]. Augusto reviewed studies on KIR genotyping in leukemias and concluded that results were conflicting and may not play major role in susceptibility [18].

KIR genes are also highly polymorphic. Currently, 1532 alleles have been identified which translate to 668 proteins. Largest number is seen in *KIR3DL3* with 228 alleles (https://www.ebi.ac.uk/ipd/kir/stats.html). This polymorphism gives rise to proteins expressed at different levels with diverse binding avidity to ligand which induces variable intensity of inhibitory signals [19]. Selection of hematopoietic stem cell donor based on KIR alleles may improve efficiency of allogeneic NK-cell-based immunotherapy nevertheless, subtyping for *KIR2DL1* alleles (*KIR2DL1*∗*001-004*) in ALL having HLA-C2 revealed no significant difference [20].

Next-generation sequencing identified unique variants in *KIR* genes among T-ALL males and controls. Interestingly, these variants were also located on different KIR genes in T-ALL male (*KIR2DL1*, *3DL2*, and *3DL3*) compared to controls (*KIR2DL4*). *KIR3DL2* was reported as a sensitive diagnostic factor and also associated with reduced disease-specific survival for CD3+ T cells in peripheral blood of Sezary syndrome (SS), a leukemic form of cutaneous T cell lymphoma (CTCL) [21] and expressed in 65.7% CTCL skin biopsies [22]. IPH4102, targeting *KIR3DL2*, is a potential checkpoint inhibitor for SS which is an aggressive disease. The variants identified here may help in differentiating responders to this immunotherapy as variants have different binding avidity to their ligands which affect intensity of inhibitory signals. There is scarce knowledge in *KIR2DL1* and *KIR3DL3* association with cancers.

The single variants identified in killer lectin-like receptors, *KLRC1/NKG2A* (rs2253849) and *KLRC2/NKG2C* (rs1141715), among T-ALL male samples here may be highly significant as these were found in almost all the patients and unique to only this group. Even though these are known variants, they have not been associated with diseases. Although previously considered to be highly conserved, polymorphism in *NKG2-A* and *NKG2-C* genes has been reported [23]. *NKG2A* is a critical checkpoint in chronic HCV infection which induces NK exhaustion which, however, can be reversed by antibody blocking [24]. To our knowledge, this is the first report on these polymorphisms in T-ALL. However, it is still unknown whether these variants are restricted to only T-ALL males or include females.

There was a significant degree of diversity within the *LILRB1* locus compared to the other genes. Many variants overlapped between T cell male and healthy male and female controls, but many others were unique to the groups. High variability in *LILRB1* was also observed in another gene sequencing study revealing 85 SNPs and 10 different alleles in healthy donors [25]. *LILRB1* is an inhibitory receptor expressed on various hematopoietic cells including NK cells. Tumor cells may use HLA-G, ligand for *ILIRB1* to inhibit NK cells. Several members of *LILRB* family are expressed by neoplastic B cells and T cell ALL which may directly regulate cancer development and relapse [26]. Our results may provide additional information in this area.

In contrast, very few or no polymorphism was detected in the other genes including the *KLRK1*/*NKG2D* ligands. These stress-associated molecules are upregulated on damaged or transformed cells to attract immune cells, especially NK cells. Previous studies observed that polymorphism in *NKG2D* ligand genes was associated with susceptibility to various diseases and disease severity including cancers and transplantation outcome. *ULBP/RAET1* genes were less polymorphic than MIC genes [27].

Copy number variations (CNVs) are present in human population with high frequency and potentially explain more variation than SNP. CNVs are both inherited and de novo in origin. CNVs range from 1Kb to several Mb in size and account for approximately 13% of the human genome [28]. A genome-wide copy number alteration study detected changes in 91.7% of 265 patients with ALL, with losses more common than gain. This allowed prediction of treatment outcome [29]. Of the 62 NK-associated genes examined here, deletions were observed in only two genes (*CD69* and *KLRD1*), while seven others (*LAIR2*, *KLRC1*, *NCR1*, *NCR3*, *KLRC2*, *C3*, and *C4*) were amplified mainly KLR genes. Our results support observation in the COSMIC Cell Lines Project (http://cancer.sanger.ac.uk) where amplifications of *LAIR2*, *KLRC1*, *NCR1*, *NCR3*, *KLRC2*, *C3*, and *C4* were reported in various cancer cell lines such as lung carcinoma, adenocarcinoma, gastric choriocarcinoma, and pancreatic cancer cell lines. Deletion of *CD69* was reported in non-small-cell lung cancer and lymph node metastasis. Nevertheless, CNVs in all these cancer cell lines did not give any effects at gene expression level [30]. Copy number loss in *KLRD1* was seen in melanoma and associated with lower T cell infiltrate and poor prognosis [31] which may have relevance to the patients here.

Deletion of *KLRD1* and *CD69* detected by NGS here was supported by quantitative PCR method. KLDR1 (CD94) pair with several NKG2 proteins with inhibitory (NKGA) as well as activating function (NKG2C and NKG2E). Nevertheless, CD94-deficient NK cells develop normally and were able to kill target cells suggesting that this gene is dispensable in NK cell maturation and function [32]. CD69 is highly upregulated in all immune cells in response to viral infection. On the other hand, CD69-deficient mice enhances NK cell response to increase protection from viral infection [33]. The effect of deletion of these genes in T-ALL is still unclear.

Copy number amplification of activation receptors, *KLRC2*, *KLRC4*, and *NCR3*, was significantly higher in female B-ALL compared to males. This implied a higher inflammatory level and a “hot” cancer which may be associated with better response.

Gene amplifications or deletions are expected to lead to gain or loss in function which eventually lead to harmful or beneficial phenotypic effects. However, direct correlations to gene and protein expressions were not always achievable. A global gene copy number variation in a mice model, revealed a weak yet significant positive correlation of copy number with gene expression [34]. Correlation of CNV to mRNA expression here was also weak.

In general, methylation index was higher in ALL than normal controls. Gao et al. showed dense methylation of CpG islands in promoter of *KIR2DL1* and *KIR2DL2/L3* correlated with very low presence of these molecules on the cell surface [35]. In fact, mRNA expressions of KIR genes observed here were low in bone marrow samples of ALL patients compared to normal peripheral blood samples and were also poorly correlated. On the other hand, infiltration of NK cells into the bone marrow of ALL patients may have been low, and thus, mRNA from these cells could not be extracted. Furthermore, normal bone marrow samples were not available as controls and thus comparison could not be made.

In the methylation index among normal controls, *2DL1*, *2DS2*, and *2DS4* were significantly higher in female. Other than the common X-chromosome inactivation in female, gender dimorphism in DNA methylation has been described in several studies showing different DNA methylation patterns in genome-wide study. Significantly lower DNA methylation level was observed in male due to lower DNA methyltransferase activity in preoptic area of brain region [36]. No earlier report on DNA methylation among gender has been reported for the KIR genes.

In contrast, a general increased methylation index (MI) was detected in male B-ALL and T-ALL compared to normal in the KIR genes analysed. Similarly, higher methylation index in *3DL2* and *2DS2* was seen in male B-ALL compared to female. No variation from this pattern was observed in inhibitory compared to activation KIR genes. The exception is *KIR2DL4*, which is constitutively expressed and reportedly not affected by DNA demethylation [37].

Costello et al. found that the expression of inhibitory *KIR3DL1* signal was downregulated in NK cell-type lymphoproliferative disease of granular lymphocyte due to methylation of the promoter [38]. Involvement of epigenetic mechanisms in regulation of *KIR2DL2* and *KIR2DL4* was observed by their upregulation and downregulation of gene expressions as the consequences of promoter demethylation and methylation [39].

DNA methylation is catalyzed by DNA-methyl-transferases (DNMTs) by the addition of a methyl-group on cytosines in the 5th position. No genetic change was observed in this gene here.

Very few reports are available on mRNA expression of NK cell receptors in acute leukemia. Low gene expression of *NCR1* on fresh and cultured NK cells as expected affected the natural cytotoxicity against target cells [40]. Downregulated NCR expression in acute myeloid leukemia patients [41, 42] also impaired effector function against autologous blast and K562 target [42]. The absence of NKG2D and other NCR ligands on leukemic blasts enabled escape from NK cell surveillance [43].

In contrast, significant increase surface expression of inhibitory receptor, *KLRC1*, in AML patients affected CD107a degranulation, TNF-*α*, and IFN-*γ* secretions that predict failure to achieve remission in AML [42].

It is generally assumed that male and female respond similarly to therapy. There is much evidence that the immune system of the different sexes responds differently to stimulation. Systematic reviews examined overall survival (OS) of male and female cancer patients given immune checkpoint inhibitors. Conforti et al. combined randomized controlled trials of immune checkpoint inhibitors (ipilimumab, tremelimumab, nivolumab, or pembrolizumab) and showed that while patients benefited from immunotherapy, the magnitude of benefit is sex dependent [44]. While observations differ, Ye et al. suggested that simply pooling different clinical trials is insufficient. Molecular profiling of individuals with immune checkpoint blockade (ICB) treatment for tumor mutation burden (TMB), individual gene mutation (PBRM1, BRCA2), T cell-inflamed gene expression profile (GEP), neoantigen load, cytolytic activity (CYT), and protein expression of checkpoints (CTLA-4, PD-L1, and PD-L2) revealed sex not only affected OS in different cancers but was also bias in the immune features across multiple cancer types [45]. Thus, evidence on sex influence in immunotherapy studies remains necessary.

## 5. Conclusion

Our results show that ALL subtypes and sex differ in mechanism of potential NK cell suppression. T-ALL male formed a relatively homogeneous group in carrying a single unique variant in *KLRC1* and *KLRC2* genes. Furthermore, there was preference for selected variants in several inhibitory KIR including *KIR3DL2*, an active framework gene. In addition, two major genes, *CD69* and *KLRD1*, in NK cells were deleted. Both B-ALL and T-ALL males were highly methylated for the majority of KIR genes and mRNA expressions for the genes were low. B-ALL female on the other hand were associated with increased gene amplification of activation genes (*KLRC2*, *KLRC4*, and *NCR3*) than males. Thus, this study provided preliminary data on potential mechanisms of NK inhibitory and activation receptors alteration in ALL subsets which may be suitable as target therapy. These should be confirmed in larger sample size and include T-ALL female samples.

## Figures and Tables

**Table 1:** Small variants with protein effects in NK cell genes shared between study groups.

Gene	T-ALL and male^∗^	T-ALL and female^∗^	Male^∗^ and female^∗^	All groups	T-ALL only	Male^∗^ only	Female^∗^ only
*KIR2DL1*	0	2	0	0	1	0	0
*KIR2DL3*	0	2	0	0	0	0	2
*KIR2DL4*	3	1	2	5	0	2	5
*KIR2DS4*	2	0	0	0	0	0	6
*KIR3DL2*	1	0	0	2	3	0	0
*KIR3DL3*	0	0	0	0	5	0	0
*KLRC1*	0	0	0	0	1	0	0
*KLRC2*	0	0	0	0	1	1	0
*KLRC3*	0	1	0	5	1	1	1
*KLRD1*	0	1	0	0	0	0	0
*NCR2*	0	2	0	0	0	0	0
*NCR1*	0	1	0	0	0	0	0
*LILRB1*	9	8	5	49	11	10	8
Total	15	18	7	61	23	14	22

^∗^Normal control.

**Table 2:** Small variants present exclusively in T-ALL patients (*n* = 6). Fisher's exact test to test associations between groups. *p* < 0.05 was considered significant.

Gene	Start	End	Reference	Variant allele	Variant type	Allele frequency	dbSNP match	dbSNP ID	Consequence	Amino acid change	*p* value
*KIR2DL1*	55294931	55294931	T	C	Substitution	0.33	Known	rs670771	Splice site		0.071
*KIR3DL2*	55328993	55328993	G	C	Substitution	0.50	Known	rs1142881	Nonsynonymous	L->F	0.024
*KIR3DL2*	55333275	55333275	G	T	Substitution	0.25	Known	rs35974949	Nonsynonymous	W->L	0.167
*KIR3DL2*	55378083	55378083	T	C	Substitution	0.25	Known	rs58413124	Nonsynonymous	L->P	0.167
*KIR3DL3*	55237603	55237603	G	A	Substitution	0.33	Known	rs11575927	Nonsynonymous	R->H	0.167
*KIR3DL3*	55237677	55237677	C	T	Substitution	0.25	Known	rs2075732	Nonsynonymous	R->W	0.167
*KIR3DL3*	55239223	55239223	G	A	Substitution	0.58	Known	rs270790	Nonsynonymous	V->I	0.024
*KIR3DL3*	55246731	55246731	C	T	Substitution	0.33	Known	rs602444	Nonsynonymous	H->Y	0.071
*KIR3DL3*	55246741	55246741	T	C	Substitution	0.33	Known	rs662386	Nonsynonymous	V->A	0.071
*KLRC1*	10603670	10603670	T	C	Substitution	1.00	Known	rs2253849	Nonsynonymous	N->S	0.005
*KLRC2*	10587111	10587111	A	G	Substitution	0.83	Known	rs1141715	Nonsynonymous	F->S	0.005
*KLRC3*	10573093	10573095	CCA	CGG	Substitution	0.41	Overlap	rs2682491, rs34195537, and rs2682490	Nonsynonymous	W->P	0.024
*LILRB1*	55143395	55143395	T	G	Substitution	0.41	Known	rs370374304	Nonsynonymous	I->S	0.024
*LILRB1*	55144708	55144711	ACAG	ACTC	Substitution	0.07	Overlap	rs199642118, rs61739175, and rs61739176	Nonsynonymous	Q->L	0.600
*LILRB1*	55142479	55142481	CCG	CTG	Substitution	0.07	Overlap	rs199604382, rs114930141	Exonic		0.600
*LILRB1*	55143183	55143183	T	C	Substitution	0.50	Known	rs368715947	Exonic		0.005
*LILRB1*	55144167	55144169	CCG	CCT	Substitution	0.07	Overlap	rs1128644, rs377724258, and rs192288587	Exonic		0.600
*LILRB1*	55148726	55148726	T	C	Substitution	0.50	Novel		Exonic		0.005
*LILRB1*	55148743	55148745	CCG	CTG	Substitution	0.07	Overlap	rs190292343	Exonic		0.600
*LILRB1*	55148889	55148891	TCG	TCA	Substitution	0.07	Novel		Exonic		0.600
*LILRB1*	55148941	55148941	T	A	Substitution	0.50	Novel		Exonic		0.005
*LILRB1*	55144773	55144773	G	A	Substitution	0.50	Novel		Splice site		0.005
*LILRB1*	55142479	55142481	CCG	CTG	Substitution	0.07	Overlap	rs199604382, rs114930141	Stop gained	R->Stop	0.600

## Data Availability

Data is available upon request. Please contact maha@upm.edu.my.

## Supplementary Materials

Supplementary Materials Table S1: demography ALL patients and normal controls. Table S2: selected natural killer cell associated genes for next-generation sequencing. Table S3: nucleotide sequence of each of the SureSelectXT indexes for Illumina sequencing. Table S4: PCR programme for amplification of index tags by posthybridization amplification. Table S5: primer sequences to determine copy number variation using quantitative PCR. Table S6: primer sequences of genes for methylation specific quantitative PCR. Table S7: primer sequences for gene expression using quantitative PCR. Table S8: small variants present exclusively in normal male controls (*n* = 4). Fisher's exact test was used to test the association between groups. Table S9: small variants present exclusively in normal female controls (*n* = 4). Fisher's exact test was used to test the association between groups.
