# Supplementary material for: Differential Regulation of NK Cell Receptors in Acute Lymphoblastic Leukemia
Source: J Immunol Res. 2022 May 23;2022:7972039. doi: 10.1155/2022/7972039 (PMC9150999; doi:10.1155/2022/7972039)
Supplement: Supplementary Materials — Table S1: demography ALL patients and normal controls. Table S2: selected natural killer cell associated genes for next-generation sequencing. Table S3: nucleotide sequence of each of the SureSelectXT indexes for Illumina sequencing. Table S4: PCR programme for amplification of index tags by posthybridization amplification. Table S5: primer sequences to determine copy number variation using quantitative PCR. Table S6: primer sequences of genes for methylation specific quantitative PCR. Table S7: primer sequences for gene expression using quantitative PCR. Table S8: small variants present exclusively in normal male controls (n = 4). Fisher's exact test was used to test the association between groups. Table S9: small variants present exclusively in normal female controls (n = 4). Fisher's exact test was used to test the association between groups. [file 7972039.f1.docx]

**SUPPLEMENTARY**

Supplementary Table S1: Demography ALL patients and normal controls.

|  | **Next-generation sequencing** | | **Copy number variation** | | |
| --- | --- | --- | --- | --- | --- |
|  | **T-ALL**  **(n=6)** | **NC**  **(n=8)** | **T-ALL**  **(n=10)** | **B-ALL**  **(n=24)** | **NC**  **(n=24)** |
| **Ethnic group** |  |  |  |  |  |
| Malay | 6 | 8 | 9 | 19 | 13 |
| Chinese | 0 | 0 | 0 | 2 | 10 |
| Indian | 0 | 0 | 1 | 1 | 0 |
| Others | 0 | 0 | 0 | 2 | 1 |
| **Gender** |  |  |  |  |  |
| Male | 6 | 4 | 10 | 12 | 12 |
| Female | 0 | 4 | 0 | 12 | 12 |
| **Age (year)** |  |  |  |  |  |
| Average (range) | 27.5  (15-38) | 26.6  (25-29) | 26.0  (4-75) | 26.5  (4-67) | 27.1  (20-51) |

Supplementary Table S2: Selected natural killer cell associated genes for next-generation sequencing

| **Genes** | | **No** | **Official symbols** | **Gene names** |
| --- | --- | --- | --- | --- |
| **KIR Genes**  immunoglobulin superfamily,  **LRC (leukocyte receptor complex)** | **Inhibition** | 1 | KIR2DL1 | killer cell immunoglobulin-like receptor, two domains, long cytoplasmic tail, 1 |
|  |  | 2 | KIR2DL2 | killer cell immunoglobulin-like receptor, two domains, long cytoplasmic tail, 2 |
|  |  | 3 | KIR2DL3 | killer cell immunoglobulin-like receptor, two domains, long cytoplasmic tail, 3 |
|  |  | 4 | KIR2DL5A | killer cell immunoglobulin-like receptor, two domains, long cytoplasmic tail, 5A |
|  |  | 5 | KIR3DL1 | killer cell immunoglobulin-like receptor, three domains, long cytoplasmic tail, 1 |
|  |  | 6 | KIR3DL2 | killer cell immunoglobulin-like receptor, three domains, long cytoplasmic tail, 2 |
|  |  | 7 | KIR3DL3 | killer cell immunoglobulin-like receptor, three domains, long cytoplasmic tail, 3 |
|  | **Activation** | 8 | KIR2DS1 | killer cell immunoglobulin-like receptor, two domains, short cytoplasmic tail, 1 |
|  |  | 9 | KIR2DS2 | killer cell immunoglobulin-like receptor, two domains, short cytoplasmic tail, 2 |
|  |  | 10 | KIR2DS3 | killer cell immunoglobulin-like receptor, two domains, short cytoplasmic tail, 3 |
|  |  | 11 | KIR2DS4 | killer cell immunoglobulin-like receptor, two domains, short cytoplasmic tail, 4 |
|  |  | 12 | KIR2DS5 | killer cell immunoglobulin-like receptor, two domains, short cytoplasmic tail, 5 |
|  |  | 13 | KIR3DS1 | killer cell immunoglobulin-like receptor, three domains, short cytoplasmic tail, 1 |
|  | **I/A*** | 14 | KIR2DL4 | killer cell immunoglobulin-like receptor, two domains, long cytoplasmic tail, 4 |
|  |  |  |  |  |
|  | **Pseudogenes** | 15 | KIR2DP1 | killer cell immunoglobulin-like receptor, two domains, pseudogene 1 |
|  |  | 16 | KIR3DP1 | killer cell immunoglobulin-like receptor, three domains, pseudogene 1 |
| **Natural Cytotoxicity Receptors (NCR)**  immunoglobulin  superfamily | **Activating** | 17 | NCR1 | natural cytotoxicity triggering receptor 1(NKp46, CD335); expressed on resting and activated cells [1] |
|  |  | 18 | NCR2 | natural cytotoxicity triggering receptor 2 (NKp44, CD336), expressed only on activated cells[1] |
|  |  | 19 | NCR3 | natural cytotoxicity triggering receptor 3(NKp30, CD337); expressed on resting and activated cells[1] |
| **Leukocyte Immunoglobulin-like receptors** | **Inhibitory** | 20 | LILRB1 | leukocyte immunoglobulin-like receptor, subfamily B (inhibitory) |
|  |  | 21 | LAIR1 | leukocyte-associated immunoglobulin-like receptor 1 (CD305), soluble receptor regulates LAIR2 |
|  |  | 22 | LAIR2 | leukocyte-associated immunoglobulin-like receptor 2 (CD306), soluble receptor inhibitory |
|  | **Activating** | 23 | CD226 | CD226 molecule (DNAM1; ligand of human CD226 was CD155 (nacl-5, PVR) and CD112 (nicotine-2) which are widely expressed including cancer) activating [2] |
| C-type lectin-like NK receptors  **NKC (natural killer gene complex)**  **KLR receptors** | **Inhibitory** | 24 | KLRB1 (CLEC5B) | killer cell lectin-like receptor subfamily B, member 1 inhibitory |
|  |  | 25 | KLRC1 (NKG2A/NKG2B) | killer cell lectin-like receptor subfamily C, member 1, CD94 (inhibitory) exhaustion molecule virus [3]  NKG2B, alternative mRNA splice products of NKG2A (protein) |
|  |  | 26 | KLRC3 (NKG2E/H) | killer cell lectin-like receptor subfamily C, member 3, complex with CD94; NKG2H, alternative mRNA splice products of NKG2E |
|  |  | 27 | KLRG1 (CLEC15A) | killer cell lectin-like receptor subfamily G, member 1 inhibitory [4] |
|  | **Activating** | 28 | KLRC2 (NKG2C) | killer cell lectin-like receptor subfamily C, member 2, complex with CD94 (activating) |
|  |  | 29 | KLRC4 (NKG2F) | killer cell lectin-like receptor subfamily C, member 4 (activating?) |
|  |  | 30 | KLRK1 (NKG2D) | killer cell lectin-like receptor subfamily K, member 1, homodimer (activating) |
|  |  | 31 | KLRF1(CLEC5C) | killer cell lectin-like receptor subfamily F, member 1, activating [5] |
|  |  | 32 | HCST | hematopoietic cell signal transducer (activation receptor) KLRK1-HCST |
|  |  | 33 | CD69 | CD69 molecule |
|  | **None** | 34 | KLRD1 (CD94) | killer cell lectin-like receptor subfamily D, member 1 |
| **KLR ligands** | | 35 | CLEC1A | C-type lectin domain family 1, member A |
|  |  | 36 | CLEC2A | C-type lectin domain family 2, member A |
|  |  | 37 | CLEC2B | C-type lectin domain family 2, member B |
|  |  | 38 | CLEC2D | C-type lectin domain family 2, member D |
|  |  | 39 | CLEC12A | C-type lectin domain family 12, member A |
| **KLRK1/NKG2D ligands** | | 40 | MICA | MHC class I polypeptide-related sequence A |
|  |  | 41 | MICB | MHC class I polypeptide-related sequence B |
|  |  | 42 | ULBP1 | UL16 binding protein 1 (ligand of NKG2D) |
|  |  | 43 | ULBP2 | UL16 binding protein 2 |
|  |  | 44 | ULBP3 | UL16 binding protein 3 |
|  |  | 45 | ULPB4/ RAET1E | retinoic acid early transcript 1E (ULBP4; NKG2D Ligand 4) |
|  |  | 46 | TNF | tumor necrosis factor |
| **Cytotoxic effector molecules** | | 47 | TNFSF9 | tumor necrosis factor (ligand) superfamily, member 9 |
|  |  | 48 | TNFSF18 | tumor necrosis factor (ligand) superfamily, member 18 |
|  |  | 49 | TNFSF13B | tumor necrosis factor (ligand) superfamily, member 13b |
|  |  | 50 | FAS | Fas (TNF receptor superfamily, member 6) |
|  |  | 51 | FASLG | Fas ligand (TNF receptor superfamily, member 6) |
|  |  | 52 | GZMB | granzyme B (granzyme 2, cytotoxic T-lymphocyte-associated serine esterase 1) |
|  |  | 53 | GNLY | granulysin |
|  |  | 54 | GZMM | granzyme M (lymphocyte met-ase 1) |
|  |  | 55 | GZMA | granzyme A (granzyme 1, cytotoxic T-lymphocyte-associated serine esterase 3) |
|  |  | 56 | GZMH | granzyme H (cathepsin G-like 2, protein h-CCPX) |
|  |  | 57 | GZMK | granzyme K (granzyme 3; tryptase II) |
| **Exhaustion molecules** | | 58 | SLAMF6 | SLAM family member 6 |
|  |  | 59 | SLAMF7 | SLAM family member 7 CD319 (SLAMF7) expression associated with T cell exhaustion surface markers [6] |
|  |  | 60 | CD244 | CD244 molecule, natural killer cell receptor 2B4 (CD244 is a Signaling Lymphocyte Activation Molecule (SLAM) family immunoregulatory receptor; potential therapeutic target; immune exhaustion (inhibitory) [7] |
|  |  | 61 | TBX21 | T-box 21 (The T-box transcription factors, T-bet and Eomes, control genes crucial for the effector functions of NK cells; IFN-γ production and cell-mediated cytotoxicity.) |
| **Others** | | 62 | DNMT3A | DNA (cytosine-5-)-methyltransferase 3 alpha |
|  |  |  |  |  |

**^I/A*-Inhibitory/activating^**

**References**

1. Paul, S., & Lal, G. (2017). The molecular mechanism of natural killer cells function and its importance in cancer immunotherapy. *Frontiers in Immunology*, **8**(**SEP**). doi:10.3389/fimmu.2017.01124
2. Zhang Z, Wu N, Lu Y, Davidson D, Colonna M, Veillette A. DNAM-1 controls NK cell activation via an ITT-like motif. *J Exp Med* (2015) 212:2165–2182. doi:10.1084/jem.20150792.
3. Zhang C, Wang X mei, Li S ran, Twelkmeyer T, Wang W hong, Zhang S yuan, Wang S feng, Chen J zheng, Jin X, Wu Y zhang, et al. NKG2A is a NK cell exhaustion checkpoint for HCV persistence. *Nat Commun* (2019) 10: doi:10.1038/s41467-019-09212-y.
4. Müller-durovic B, Lanna A, Covre LP, Mills RS. Killer Cell Lectin-like Receptor G1 ( KLRG1 ) inhibits NK cell function through activation of AMP-activated Protein Kinase. *J Immunol* (2017) 197:2891–2899. doi:10.4049/jimmunol.1600590.Killer
5. Kuttruff S, Koch S, Kelp A, Pawelec G, Rammensee HG, Steinle A. NKp80 defines and stimulates a reactive subset of CD8 T cells. *Blood* (2009) 113:358–369. doi:10.1182/blood-2008-03-145615
6. Awwad MHS, Mahmoud A, Bruns H, Echchannaoui H, Kriegsmann K, Lutz R, Raab MS, Bertsch U, Munder M, Jauch A, et al. Selective elimination of immunosuppressive T cells in patients with multiple myeloma. *Leuk 2021 359* (2021) 35:2602–2615. doi:10.1038/s41375-021-01172-x.
7. Agresta L, Hoebe KHN, Janssen EM. The emerging role of CD244 signaling in immune cells of the tumor microenvironment. *Front Immunol* (2018) 9:1–9. doi:10.3389/fimmu.2018.02809

Supplementary Table S3: Nucleotide sequence of each of the SureSelectXT indexes for Illumina sequencing.

| **Index number** | **Sequence** |
| --- | --- |
| 1 | ATCACG |
| 2 | CGATGT |
| 3 | TTAGGC |
| 4 | TGACCA |
| 5 | ACAGTG |
| 6 | GCCAAT |
| 7 | CAGATC |
| 8 | ACTTGA |
| 9 | GATCAG |
| 10 | TAGCTT |
| 11 | GGCTAC |
| 12 | CTTGTA |
| 13 | AAACAT |
| 14 | CAAAAG |

Supplementary Table S4: PCR programme for amplification of index tags by post-hybridization amplification.

| **Step** | **Temperature** | **Time** |
| --- | --- | --- |
| Step 1 | 98^o^C | 2 min |
| Step 2 | 98^o^C | 30 s |
| Step 3 | 57^o^C | 30 s |
| Step 4 | 72^o^C | 1 min |
| Step 5 |  | Repeat step 2 through 4 for 12 times |
| Step 6 | 72^o^C | 10 min |
| Step 7 | 4^o^C | Hold |

Supplementary Table S5: Primer sequences to determine copy number variation using quantitative PCR.

| **Target genes primer pairs** | **Sequences (5’ to 3’)** | **Product sizes (bp)** |
| --- | --- | --- |
| *CD56* forward | AGG TGG ATA AGA ACG ACG AGG^1^ | 210 |
| *CD56* reverse | TTC GCT GCT GAT GTT CCG^1^ |  |
| *NCR1* forward | GAA ATT ATG GGG CTG TTG AAT A | 116 |
| *NCR1* reverse | GTT CAT GTC CGG GAT GTA GA |  |
| *NCR3* forward | TCC ACT CTG CAC ACG TAG ATG | 131 |
| *NCR3* reverse | AAT GGA ACC CCA GAG TTC AG |  |
| *CD69* forward | ACA GAG CAG CAT CCA CTG AC | 132 |
| *CD69* reverse | AGC AGC ATG GAA TGT GAG AA |  |
| *LAIR2* forward | AGG CCA GAT TCC ACA TTG AC | 81 |
| *LAIR2* reverse | ACC ATC CAG GGG GCT TAT |  |
| *KLRC1* forward | GTT TTC GTT GCT GCC TCT TT | 96 |
| *KLRC1* reverse | AGT CCC TGA CAT CAC ACA CTG |  |
| *KLRC2* forward | ACA GGC CAG CAA ACT CTC TT | 92 |
| *KLRC2* reverse | GCC ATT GTC CTG AGG AGT G |  |
| *KLRC3* forward | TCC TGT TCG GTT CCT GAA AT | 130 |
| *KLRC3* reverse | CAC ATC ACA CAG CTG CAG AG |  |
| *KLRC4* forward | GGC CAG CAA ACT CTT TCT TC | 91 |
| *KLRC4* reverse | GCC ATT GTC CTG AGG AGT G |  |
| *KLRD1* forward | GGC TTC ATC TTG CTA ATG TGT AA | 134 |
| *KLRD1* reverse | TCT ATG TTG GGT CCT GGA GTA A |  |

^1These primers were previously described by Weksberg et al. (2005) (68)^

Supplementary Table S6: Primer sequences of genes for methylation specific quantitative PCR.

| **Reference gene primers** | **Sequences (5’ to 3’)** | **Product sizes (bp)** |
| --- | --- | --- |
| *beta-actin* forward | TGG TGA TGG AGG AGG TTT AGT AAG T^1^ | 135 |
| *beta-actin* reverse | AAC CAA TAA AAC CTA CTC CTC CCT TAA^1^ |  |
| *KIR2DL2*-M-forward | TAG GGC GTT AAA TAA TAT TTT GTG C^2^ | 100 |
| *KIR2DL2*-M-reverse | GCC ATA CTA ACG ACC ATA AAC G^2^ |  |
| *KIR2DL2*-U-forward | GGG TGT TAA ATA ATA TTT TGT GTG T^2^ | 101 |
| *KIR2DL2*-U-reverse | CAC ACC ATA CTA ACA ACC ATA AAC AAC^2^ |  |
| *KIR2DL4*-M-forward | TTT ATT ATT TGA ATT TTA TAT GAC GT^2^ | 132 |
| *KIR2DL4*-M-reverse | ATA ACT CGA CTC GAC TAA CCG AT^2^ |  |
| *KIR2DL4*-U-forward | TTT ATT ATT TGA ATT TTA TAT GAT GT^2^ | 134 |
| *KIR2DL4*-U-reverse | CAA TAA CTC AAC TCA ACT AAC CAA T^2^ |  |
| *KIR3DL2*-M-forward | TTT TAT GTA AGG TAG AAA GAG TTT GC | 101 |
| *KIR3DL2*-M-reverse | ACA ACG CAC AAA ATA TTA TTT AAC G |  |
| *KIR3DL2*-U-forward | GGT TTT ATG TAA GGT AGA AAG AGT TTG T | 102 |
| *KIR3DL2*-U-reverse | CAA CAC ACA AAA TAT TAT TTA ACA CC |  |
| *KIR3DL3*-M-forward | TAA TTT TTA GGA AGT TAT GTT TCG T | 180 |
| *KIR3DL3*-M-reverse | CCG ATA CAA ACA AAC GAC TAC G |  |
| *KIR3DL3*-U-forward | TGG AAT AAT TTT TAG GAA GTT ATG TTT T | 186 |
| *KIR3DL3*-U-reverse | ACC AAT ACA AAC AAA CAA CTA CAC C |  |
| *KIR2DS2*-M-forward | TTT TAT GTA AGG TAG AAA GAG TTT GC | 101 |
| *KIR2DS2*-M-reverse | ACA ACG CAC AAA ATA TTA TTT AAC G |  |
| *KIR2DS2*-U-forward | GGT TTT ATG TAA GGT AGA AAG AGT TTG T | 102 |
| *KIR2DS2*-U-reverse | CAA CAC ACA AAA TAT TAT TTA ACA CC |  |
| *KIR2DS4*-M-forward | AGG AAG TTA TGT TTT GTT TTT GAG C | 138 |
| *KIR2DS4*-M-reverse | AAC GCA CAA AAT ATT ATT TAA CGC |  |
| *KIR2DS4*-U-forward | AAG TTA TGT TTT GTT TTT GAG TGA | 135 |
| *KIR2DS4*-U-reverse | AAC ACA CAA AAT ATT ATT TAA CAC C |  |

“M” stands for methylated where else “U” stands for unmethylated.

^1These primers were previously described by Hattermann et al. (2008) [1]^

^2These primers were previously described by Liu et al. (2009) [2]^

**References**

1. Hattermann K, Mehdorn HM, Mentlein R, Schultka S, Held-Feindt J. A methylation-specific and SYBR-green-based quantitative polymerase chain reaction technique for O6-methylguanine DNA methyltransferase promoter methylation analysis. *Anal Biochem* (2008) 377:62–71. doi:10.1016/j.ab.2008.03.014
2. 70. Liu Y, Kuick R, Hanash S, Richardson B. DNA methylation inhibition increases T cell KIR expression through effects on both promoter methylation and transcription factors. *Clin Immunol* (2009) 130:213–224. doi:10.1016/j.clim.2008.08.009

Supplementary Table S7: Primer sequences for gene expression using quantitative PCR.

| **Reference gene primers** | **Sequences (5’ to 3’)** | **Product sizes (bp)** |
| --- | --- | --- |
| *GAPDH* forward | GTC AAG GCT GAG AAC GGG AAG | 215 |
| *GAPDH* reverse | CCT TCG CCG TCT CCA TAG |  |
| *NCR1* forward | AGA GGG TGG GTG TGT CAT | 131 |
| *NCR1* reverse | CTA CAT CCC GGA CAT GAA CTC |  |
| *NCR2* forward | CCA GAT TGT GAA TCG AGA GGT C | 102 |
| *NCR2* reverse | AAG AAA GGC TGG TGT AAG GAG |  |
| *LILRB1* forward | CGT TCC CCG TCC TTA TAC AG | 102 |
| *LILRB1* reverse | AGT GCA GCC AGG TCC TAT |  |
| *KLRC1*forward | GTT TTC GTT GCT GCC TCT TTG | 141 |
| *KLRC1* reverse | CCT CTC CAC TAA AGG ATG TGT G |  |
| *KLRC2* forward | CGA TTT ACT TGT AGC ACT GCA C | 146 |
| *KLRC2* reverse | GCC AGC ATT TTA CCT TCC TCA |  |
| *KLRC3* forward | CAT GGA TGA TGA CTG CTG TTA C | 125 |
| *KLRC3* reverse | GCC TGT GCT TCA AAG AAC TCT |  |
| *KLRC4* forward | GCT CCA GTA CTC CAA TAC AAG G | 143 |
| *KLRC4* reverse | GAA TGA CAA GAC ATA TCA CTG CAA |  |
| *KLRD1* forward | CAC TGT AAG AGA GTC CAA TCC A | 143 |
| *KLRD1* reverse | CAG TGA ACA GAA AAC TTG GAA CG |  |
| *GP6* forward | GCA TAT CCG GAC CAG GTT G | 107 |
| *GP6* reverse | CTG ACC GTC TCA TTC ACA CAA C |  |
| *KIR3DL2* forward | TGT CCT GAC CAC CCA TGA G | 94 |
| *KIR3DL2* reverse | ACC GGC AGC ACC ATG TC |  |

Supplementary Table S8: Small variants present exclusively in normal male controls (n=4). Fisher’s exact test was used was used to test association between groups. p<0.05 was considered significant.

| **Gene** | **Start** | **End** | **Reference** | **Variant allele** | **Variant type** | **. Allele frequency** | **dbSNP**  **match** | **dbSNP**  **ID** | **Consequence** | **Amino acid**  **change** | **P-value**  **T-ALL** | **P-value female** |
| --- | --- | --- | --- | --- | --- | --- | --- | --- | --- | --- | --- | --- |
| *KIR2DL4* | 55333275 | 55333275 | G | T | Substitution | 0.38 | Known | rs35974949 | Non-synonymous | W->L | 0.133 | 0.214 |
| *KIR2DL4* | 55349273 | 55349273 | C | A | Substitution | 0.38 | Known | rs1130492 | Non-synonymous | P->T | 0.133 | 0.214 |
| *KLRC2* | 10588581 | 10588581 | T | C | Substitution | 0.38 | Known | rs28403159 | Non-synonymous | N->S | **0.033** | 0.071 |
| *KLRC3* | 10571672 | 10571672 | T | C | Substitution | 0.38 | Novel | - | Non-synonymous | N->S | **0.033** | 0.071 |
| *LILRB1* | 55143198 | 55143201 | CCGC | CTGG | Substitution | 0.27 | Overlap | rs369637637,  rs138410838,  rs142396802,  rs10418732 | Non-synonymous | R->W | **0.033** | 0.071 |
| *LILRB1* | 55143199 | 55143199 | C | T | Substitution | 0.13 | Known | rs138410838 | Non-synonymous | R->C | 0.400 | 0.500 |
| *LILRB1* | 55143393 | 55143395 | CAT | CAG | Substitution | 0.13 | Overlap | rs370374304 | Non-synonymous | I->S | 0.400 | 0.500 |
| *LILRB1* | 55144010 | 55144010 | A | G | Substitution | 0.50 | Novel | - | Non-synonymous | N->D | **0.005** | **0.014** |
| *LILRB1* | 55144206 | 55144208 | TCG | TGG | Substitution | 0.27 | Overlap | rs372158181,  rs61737955,  rs139074994 | Non-synonymous | IA->MA | **0.033** | 0.071 |
| *LILRB1* | 55144567 | 55144567 | A | C | Substitution | 0.50 | Novel | - | Non-synonymous | Q->H | **0.005** | **0.014** |
| *LILRB1* | 55142540 | 55142542 | ACG | ACA | Substitution | 0.11 | Overlap | rs370113006,  rs368151169 | Exonic | - | 0.400 | 0.500 |
| *LILRB1* | 55148074 | 55148076 | GCG | GCA | Substitution | 0.33 | Overlap | rs61743117,  rs62133433 | Exonic | - | **0.005** | **0.014** |
| *LILRB1* | 55148905 | 55148908 | GTCA | GGCA | Substitution | 0.10 | Novel | - | Exonic | - | 0.4 | 0.5 |
| *LILRB1* | 55148925 | 55148925 | A | G | Substitution | 0.25 | Novel | - | Exonic | - | 0.133 | 0.214 |

Supplementary Table S9: Small variants present exclusively in normal female controls (n=4). Fisher’s exact test was used was used to test association between groups. p<0.05 was considered significant.

| **Gene Symbol** | **Start** | **End** | **Reference** | **Variant allele** | **Variant type** | **Allele**  **Frequency** | **dbSNP**  **match** | **dbSNP**  **ID** | **Consequence** | **Amino acid change** | **P-value T-ALL** | **P-value male** |
| --- | --- | --- | --- | --- | --- | --- | --- | --- | --- | --- | --- | --- |
| *KIR2DL3* | 55253521 | 55253521 | C | G | Substitution | 0.50 | Known | rs35719984 | Non-synonymous | Q->E | **0.033** | 0.071 |
| *KIR2DL3* | 55263194 | 55263194 | G | C | Substitution | 0.50 | Known | rs2966879 | Non-synonymous | C->S | **0.033** | 0.071 |
| *KIR2DL4* | 55317439 | 55317439 | C | T | Substitution | 0.25 | Known | rs2075769 | Non-synonymous | P->L | 0.133 | 0.214 |
| *KIR2DL4* | 55341575 | 55341575 | G | C | Substitution | 0.25 | Known | rs1130513 | Non-synonymous | E->Q | 0.133 | 0.214 |
| *KIR2DL4* | 55359436 | 55359436 | G | C | Substitution | 0.38 | Known | rs78931759 | Exonic | - | **0.033** | 0.071 |
| *KIR2DL4* | 55359444 | 55359444 | G | C | Substitution | 0.38 | Known | rs17173061 | Exonic | - | **0.033** | 0.071 |
| *KIR2DL4* | 55359879 | 55359879 | G | C | Substitution | 0.38 | Known | rs622363 | Exonic | - | **0.033** | 0.071 |
| *KIR2DS4* | 55349264 | 55349264 | G | A | Substitution | 0.25 | Known | rs115093949 | Non-synonymous | G->S | 0.133 | 0.214 |
| *KIR2DS4* | 55350948 | 55350948 | A | T | Substitution | 0.25 | Known | rs4806589 | Non-synonymous | T->S | 0.133 | 0.214 |
| *KIR2DS4* | 55350979 | 55350979 | A | G | Substitution | 0.25 | Known | rs4806590 | Non-synonymous | K->R | 0.133 | 0.214 |
| *KIR2DS4* | 55351022 | 55351022 | A | C | Substitution | 0.25 | Known | rs4806591 | Non-synonymous | N->H | 0.133 | 0.214 |
| *KIR2DS4* | 55358655 | 55358655 | A | G | Substitution | 0.38 | Known | rs1654643 | Non-synonymous | Y->C | **0.033** | 0.071 |
| *KIR2DS4* | 55358734 | 55358734 | G | A | Substitution | 0.38 | Known | rs1743319 | Non-synonymous | D->N | **0.033** | 0.071 |
| *KLRC3* | 10571663 | 10571663 | G | A | Substitution | 0.38 | Novel | - | Non-synonymous | P->L | **0.033** | 0.071 |
| *LILRB1* | 55142745 | 55142748 | CACG | CACA | Substitution | 0.10 | Novel | - | Non-synonymous | HV->HM | 0.400 | 0.500 |
| *LILRB1* | 55143174 | 55143176 | TTA | TTT | Substitution | 0.20 | Overlap | rs370310543 | Non-synonymous | Y->F | 0.133 | 0.214 |
| *LILRB1* | 55143176 | 55143176 | A | T | Substitution | 0.25 | Novel | - | Non-synonymous | Y->F | 0.133 | 0.214 |
| *LILRB1* | 55143557 | 55143557 | C | G | Substitution | 0.13 | Novel | - | Non-synonymous | S->W | 0.400 | 0.500 |
| *LILRB1* | 55144206 | 55144208 | TCG | TCA | Substitution | 0.28 | Overlap | rs372158181,  rs61737955,  rs139074994,  rs201552016 | Non-synonymous | IA->IT | **0.033** | 0.071 |
| *LILRB1* | 55143060 | 55143060 | A | G | Substitution | 0.50 | Novel | - | Exonic | - | **0.005** | **0.014** |
| *LILRB1* | 55144066 | 55144066 | A | AG | Insertion | 0.13 | Overlap | rs375561323 | Exonic | - | 0.400 | 0.500 |
| *LILRB1* | 55142479 | 55142481 | CCG | CTG | Substitution | 0.11 | Overlap | rs199604382,  rs114930141 | Stop gained | R->Stop | 0.400 | 0.500 |
